# Causal narratives in public health: the difference between mechanisms of aetiology and mechanisms of prevention in non‐communicable diseases

**DOI:** 10.1111/1467-9566.12621

**Published:** 2017-10-11

**Authors:** Michael P. Kelly, Federica Russo

**Affiliations:** ^1^ Primary Care Unit Institute of Public Health University of Cambridge UK; ^2^ Department of Philosophy University of Amsterdam The Netherlands

**Keywords:** public health, non‐communicable disease, social practice, behaviour change, alcohol

## Abstract

Research in the health sciences has been highly successful in revealing the aetiologies of many morbidities, particularly those involving the microbiology of communicable disease. This success has helped form a narrative to be found in numerous public health documents, about interventions to reduce the burden of non‐communicable diseases (e.g., obesity or alcohol related pathologies). These focus on tackling the purported pathogenic factors causing the diseases as a means of prevention. In this paper, we argue that this approach has been sub‐optimal. The mechanisms of aetiology and of prevention are sometimes significantly different and failure to make this distinction has hindered efforts at preventing non‐communicable diseases linked to diet, exercise and alcohol consumption. We propose a sociological approach as an alternative based on social practice theory. (A virtual abstract for this paper can be found at: https://www.youtube.com/channel/UC_979cmCmR9rLrKuD7z0ycA).

## Introduction

This paper considers the way that simple causal narratives dominate political and policy discourse about the prevention of non‐communicable diseases (NCDs). While we consider, specifically, the case of English policies, we think analogous approaches could be applied in other countries. These narratives impute mechanisms. The idea is borrowed from the aetiology of infectious or communicable disease; once you know the mechanisms you can intervene on it and prevent disease. In NCDs, instead of germs and viruses causing illness, the putative causal pathway runs from human behaviour, through risk, to disease and then on to prevention. The behaviour, its associated risks, the disease and its prevention are treated as if they are part of the same causal chain (see Department of Health 2004, 2010, Department of Health/Public Health England 2015a).

The simplified narrative acts as a heuristic, that is, a shorthand way of thinking that allows simple but inaccurate answers to complicated problems to frame decision making (Kahneman 2011). The simple narrative conceals the vital distinction between mechanisms of aetiology and mechanisms of prevention. In the following two sections 2 and 3, we argue that the mechanisms of aetiology and mechanisms of prevention must be distinguished. In the 4 and 5 sections, we propose a non‐reductionist sociological approach to health and disease, based on social practice theory and on a brief consideration of nineteenth century public health in England. Finally, in the sixth and seventh sections, we illustrate our argument with discussions about the prevention of smoking, obesity and using social practice theory show how this helps illuminate preventive approaches to alcohol misuse.

## Mechanisms of aetiology and of prevention: simple narratives

The simple causal narrative is that non‐communicable diseases (NCDs) arise from a set of well‐known risks which have been identified in epidemiological studies (McMichael 1999), particularly: smoking, overeating or not eating the right kinds of food, not taking enough exercise and drinking too much alcohol. These risks are attributed to the consequences of people's behaviours and lifestyles or their inability to make healthy choices on account of the social conditions in which they live (see Department of Health 1992, 1999, 2003, 2004, 2010; see also Kriznik 2015). Therefore, in order to reduce exposure to these risks, interventions to change behaviours and lifestyles are required. In other words, once a plausible account of the aetiology of the disease is established (the risky behaviour), it is assumed that acting on the same mechanisms (the behaviour) will reduce the burden of disease (Department of Health 1992, 1999, 2004, 2010). As we shall argue in the following section, it is sometimes not the case that intervening on the same mechanism will reduce the burden of disease.

NCDs are a global problem; they have overtaken infectious diseases as the major causes of death world‐wide (WHO 2014). The need to find a way to deal with the problem is an urgent priority in the face not only of the increasing prevalence but also the health inequalities associated with these epidemics (Ash *et al*. 2012, Beaglehole *et al*. 2012, Horton 2013, United Nations General Assembly 2011, WHO 2012). This has been called the ‘fourth epidemiological transition’, a transition believed by many to be powered by human behaviour (Buck and Frosini 2012, Kelly and Barker 2016, Mackenbach 2012). The idea of changing risky behaviours has been the driving force behind preventive efforts around the world for decades through legislation, regulation, taxation, advertising, education, information provision, health warnings, social marketing, nudging, counselling and a whole array of psychologically based techniques (Marteau *et al*. 2015).

In this view of things disease is conceptualised as the consequence of exposure to a pathogen or other preceding noxious agent. On the basis of exposure to the pathogen, within certain broad limits, the likely disease outcome can be predicted and the treatment and or prevention options can be organised (Broadbent 2011, Carter 2003). The principle is that there are causes and, more specifically, the preceding noxious causal agent in the case of non‐communicable diseases is human behaviour. This is inferred from known risks (established, broadly, via epidemiological studies). To change the effect (the burden of disease), the solution is to induce change in human behaviour.

This principle is to be found, in one form or another, in almost all documents on the topic of prevention of non‐communicable disease produced by the English Department of Health, other parts of the UK, the EU, and more broadly by WHO and the United Nations. In 2014, for example WHO stated that the contemporary epidemic of non‐communicable diseases in developed and developing societies is attributable to people's smoking, dietary habits, physical inactivity and alcohol consumption – their individual behaviours – which then become the target of preventive interventions (WHO 2014). Likewise, as noted above, policy documents from the English Department of Health and more recently of Public Health England are replete with this same simple story. This narrative has been repeated down the years. Since at least the publication of *Prevention and Health: Everybody's Business* in 1976 (Department of Health and Social Security 1976) through to the present (see Department of Health 1987, 1999, 2004, 2010) and most recent pronouncements by Public Health England on reducing alcohol misuse and childhood obesity (Department of Health/Public Health England 2015a, HM Government 2016, Public Health England 2016), the same simple story prevails.

Aaron Antonovsky called this way of thinking *pathogenesis*: seeking out the origins of illness by linking bad precursors to bad outcomes (Antonovsky 1985, 1987). He proposed the opposite as a more apposite epistemology in what he called *salutogenesis* – meaning the origins of health. We take inspiration from Antonovsky in distinguishing mechanisms that produce disease from mechanisms that produce health, such as encouraging preventive measures. These mechanisms, we submit, are not the same.

For instance, while the pathogenesis of lung cancer involves exposure to carcinogens in cigarette smoke, the dose response relationship between exposure and disease does not in itself explain how to help people to quit or how to reduce the population prevalence of smoking. The epidemiological evidence demonstrates that it should be a public health priority and what to do, namely, to reduce exposure to tobacco smoke. The biology of lung cancer however doesn't tell you how to do it. In the simple narrative the ‘should’, the ‘what’ and the ‘how’, are conflated. With tobacco control, which is a quintessentially salutogenic activity, the how involves a number of mechanisms pertaining to the social sphere, including different social practices and structural determinants of health (Blue *et al*. 2016, Holman and Borgstrom 2016, see also following section).

It is in principle a relatively straightforward matter to apply the same analytic framework to food and alcohol consumption and physical activity (see below) in order to examine the interrelations between the elements of the practices as a way of developing effective preventive strategies. The approach allows for thinking anew about the problem and of extracting the narrative from simple linear models of prevention. This, we argue, is important because although Antonovsky's ideas about salutogenesis have been very influential in some fields of health promotion and public health, pathogenesis, as he observed four decades ago, remains a dominant mode of organising health care and services and indeed prevention. We argue that the concepts of cause, risk and prediction as they apply to aetiology and prevention need to be unpicked using social practice theory to help challenge the dominant narrative and to get to better solutions for prevention.

The assumption driving the simple narrative, that is, that the aetiology of infectious disease is simple and linear, is actually empirically and medically wide of the mark. On the one hand, contemporary medicine uses highly sophisticated multifactorial, non‐linear, non‐mono‐causal accounts of the action of infective agents and indeed the aetiologies of both communicable and non‐communicable diseases are recognised to be highly complex, involving multiple interactions between social, behavioural and biological processes and factors (Kelly *et al*. 2014). On the other hand, it isn't the case that this way of thinking – the simple narrative – has not been challenged. But the ideas and the implications of a vast body of work focusing on the social determinants of health have not been translated into preventive activity (Glasgow and Schrecker 2015) in spite of the fact that it is often acknowledged in many of the documents produced by the English Department of Health and more broadly (see for example Department of Health 2010, Commission on the Social Determinants of Health 2008). The very large amount of evidence pointing to structural determinants of health and disease and patterns of inequalities is largely absent from policy programmes other than in a rhetorical role (Bonnefoy *et al*. 2007, Kelly and Doohan 2012).

The strong emphasis placed on simple accounts of individual behaviour and of its associated risks drives attention away from the broader factors at work (Glass *et al*. 2013, Glasgow and Schrecker 2015, McMichael 1999). Policy makers default to simple heuristics of individual level behaviour change because it is apparently easier and more obvious than the complex thinking required to engage whole populations, or indeed to confront those anti‐health forces whose profit margins depend on selling the population salty, fatty, sugary foods of low nutritional value, of flooding the market with cheap alcohol, or providing carbon greedy forms of private transport at the expense of walking or cycling.

For this to change, sociologists and philosophers must work with policy makers, politicians, political parties, journalists, and opinion leaders. The sociological arguments advanced here must be brought into the trade in ideas in the world of policy (Smith 2013). Presently, the sociological voice is mostly unheard in these environments. For the ideas to take root will require concerted and repeated forays into Whitehall, Westminster, the WHO and beyond. Structurally one of the most helpful things that could happen, and is something that ought to be consistently argued for, is the uncoupling of the public health policy process from the political and electoral cycle. Politicians inevitably default to quick and easy ideas because they have very little time to make their mark. The long haul that is successful public health policy (such as has been achieved in tobacco control) is considerably greater than the time from one general election to another. The uncoupling is not inconceivable – responsibility for setting interest rates was removed from the control of the Chancellor of the Exchequer and given to a committee in the Bank of England some years ago. Something akin to this would be enormously helpful and allow for a much more dispassionate and objective approach to evidence to inform policy than the current hurly burly of party political partisanship allows.

Commercial interests are not going to go away either or have a Damascene conversion. More and stronger regulation must be part of the solution but the process of establishing regulation will be far easier if it flows from the recommendations of independent scientific committees, rather than politicians. But it is more than regulation. It is about understanding the mechanisms that produce disease and the mechanisms that may reduce disease. So, for instance, food producers need to be engaged in active strategies for healthier product reformulation. Alcohol producers and advertisers will not act responsibly by being exhorted or trusted to do so. A framework for alcohol marketing and selling needs to be devised independently of the party political process. With physical activity, the infrastructure of town and country and the way it facilitates walking and cycling and provides efficient, reliable, economic and sustainable public transport should also be the focus of independent scientific assessment and recommendation.

## Dis‐assembling simple heuristics for non‐communicable diseases

Contained in the simple narrative are a set of assumptions about mechanisms of action and therefore of prevention: the behaviours involved in aetiology are either the same as, or the flip side of or opposite to, the behaviours that need to be undertaken to implement successfully, preventive activities. In this framework, the opposite of smoking is not smoking, the opposite of eating too much is eating less – and so on. Yet, there are decades of research in sociology and psychology demonstrating that the behaviours involved in the mechanisms of disease causation and the mechanisms involved in prevention of, and protection from, disease are different. For example the reasons for taking up smoking and continuing to smoke and the reasons for and engaging in the process of quitting smoking are quite different. They are not the same behaviour or a unitary behaviour or social action nor mirror images of each other (see Rosenstock 1960, 1966, Kirscht *et al*. 1966, Becker 1979 1979, Becker *et al*. 1977, Bell *et al*. 2011, Bellis *et al*. 2006, Mechanic 1962, 1972; Mechanic and Volkart 1960, Sheron *et al*. 2012, Spoth *et al*. 2008, Twigg and Moon 2013, Waters *et al*. 2010, West *et al*. 2000). Nevertheless, in contemporary English public health policy and beyond, there has been a long‐term failure to recognise this difference and to act accordingly.

Later we discuss in detail smoking and alcohol use in this respect, but let us anticipate our argument by focusing here on obesity (see Department of Health 2009). The rationale for policy is that it is important to reduce or prevent obesity because it is a risk factor for cardio‐vascular disease, certain cancers, and type 2 diabetes. The relationship between the biology of the diseases and the risk, that is, obesity, is epidemiologically very well established. The preventive efforts are designed to do something about limiting risk by reducing excessive calorie consumption. So an action A (encouraging people to consume fewer calories via an education effort like the current English ‘Change4Life Campaign’, Department of Health/Public Health England 2015b) is taken at time T1 and is expected to have an outcome B (i.e. reduced prevalence of obesity) at some point in the future at time T2. The assumption is that an accurate prediction about the outcome of action A can be made.

If there were a precise correspondence between ‘excess calorie consumption makes you put on weight’ (the risk and the cause) and ‘eating less will make you lose weight’ (the solution and the outcome), it would be possible to make an accurate prediction of the effectiveness of said intervention. But of course there isn't an exact correspondence, and therefore that prediction is not very secure. Even though it is known that if calorie consumption exceeds energy expenditure people generally do put on weight, and it is also known that reducing calorie intake will help people to lose weight, the prediction of future behavioural outcomes cannot be inferred from scientific knowledge about the consequences of excessive calorie consumption. Furthermore, educating the public about calorie consumption will not do the job either because knowledge about food, even highly accurate nutritional information, is only one (and a very small) part of the social practice of eating. In sociological terms this is obvious – in policy terms it is not (for a discussion of obesity policies, see also Russo 2012).

The error, though, is not just a failure to reflect upon eating as a cultural and social practice, but it is to assume that the knowledge at point T1 about the likely outcome of encouraging people to consume fewer calories is analogous to the knowledge of the preceding factors in the cause of bacterial and viral disease and reducing exposure to those preceding factors! In fact, in each case different types of inference and explanation are involved – a problem explored at length by philosophers since the important work of Lipton (2004) and further examined in recent approaches to evidence and causation (see e.g. Clarke *et al*. 2014, Reiss 2015). The predictions about the outcomes of interventions to encourage the consumption of fewer calories involve human and organisational behaviour – not biology – and human behaviour is not amenable to simple prediction (Blue *et al*. 2016).

This point is important. It has escaped the attention not only of policy makers but also of scholars in the field of the philosophy of mechanisms too (for an overview on the philosophy of mechanisms, see Illari and Russo 2014, and references therein, Glennan and Illari 2017). Scholars in this field have majored on complexity of disease mechanisms but not the distinction we draw here. In this literature, accounts of aetiology emphasise the causal role of certain factors for the correct or incorrect functioning of a given mechanism. Thus for instance, scientists and philosophers alike have studied in detail the functions of genes, what happens under normal circumstances, when they are suppressed, or expressed. However, as recent studies in epigenetics show, the causes of health and disease are to be found also in places other than pathogens that are reducible to biochemical factors (Kelly and Kelly 2017). This supports the view that mechanisms used to explain a given health condition are not necessarily the same as the mechanisms used to prevent such conditions occurring.

The idea that these mechanisms must be the same is perhaps inherited from the important work of Nancy Cartwright (1979), where she defends causal laws on the grounds that these are ‘effective strategies’ to obtain the sought effects. A similar view has been supported by Donald Gillies (2005a) too, who emphasises the ‘usefulness’ of causes in order to take appropriate action. While we agree with the view that mechanisms of prevention must specify causal factors, our point is that the relevant causal factors may be quite different depending on whether mechanisms of aetiology or of prevention are being scrutinized.

To return to the case of obesity, the detailed descriptions of the complexities relating to obesity were extremely well articulated in the Foresight Report (Butland *et al*. 2007). This report was sponsored by the UK Government Office for Science and examined the interrelationships between social, economic, commercial and clinical as well as behavioural factors in the origins of the rising prevalence of obesity. It considered policy options for the future and using a whole systems perspective highlighted the complexities involved. But that complexity has been for the most part eschewed in policy action driven by what amount to simple heuristics. So policy has continued down the line of encouraging people to make healthier choices, of getting the food industry to act responsibly to help in such choice making and providing information about calorie content in order that people can work out what they're eating. The relational and dynamic nature of the problem as spelt out by the Foresight Report is entirely absent.

## The potential contribution of the behavioural sciences

There is a whole range of relevant evidence about complex behaviours and social processes involved in these matters, and about which a great deal is known scientifically. There is a huge literature explaining how health‐related behaviour change operates and more importantly discussing the reasons why the outcome of these efforts at change vary widely and on the whole have shown only limited effectiveness (see Marteau *et al*. 2015). There is also a highly respected science of behaviour change which elucidates in great detail what the evidence base contains, its strengths and limitations and appropriate ways to implement it (Michie *et al*. 2014).

This evidence is the basis of guidelines developed at the National Institute for Health and Care Excellence (NICE) where evidence and data drawn from psychology and sociology are used to place behaviour change in its social and economic context (NICE 2007, 2014). The sociological study of health has made clear that health and disease are not just bio‐chemical phenomena. Instead, their aetiology and prevention are influenced by socio‐economic factors (‘structural determinants’ of health) and involve diverse social practices that may vary across time, culture, or geographic location. Ignoring this complexity has resulted in failed interventions, such as the Tamil Nadu Nutrition Project (for a discussion, see Cartwright 2011).

So, although there is a science that could be pressed into service, it is not the basis of most policy (Kelly and Barker 2016). Instead, policy typically proceeds as if risk and health related behaviour change can be linked in a simple and straightforward way. Most public health interventions therefore avoid the complexity of the psychological or the sociological sciences and default to an easier and simpler model. Politicians and policy makers fall back on simple solutions and heuristics and consequently fail to make the most of what is known (Kahneman 2011).

Later we provide more details about how studying the social dimension of health and disease can help identify the mechanisms of disease and the mechanisms of prevention. Lessons from the studies that we here only summarily present should be systematically employed not only for the production of guidelines (see e.g. NICE mentioned above) but also in the development of policy documents, and especially in the implementation of policy interventions.

## The complex history of nineteenth century public health in Britain

Until now we have discussed how the simple linear causal narrative pervades present day public health policy. Yet, it is worth asking what the origins of this simple narrative are. Arguably, the narrative draws upon commonplace ideas about the provision of clean water and sewage disposal and the discovery of germs in the nineteenth century, as paradigmatic examples of ‘simple’ public health interventions and of ‘clearly identifiable’ (biological) causal factors.

In Britain, the fear of cholera was a more or less continuous feature of Victorian life (Hamlin 2009). In 1884, in Germany, Koch isolated the bacteria responsible for cholera. A few years later, in 1891, after the death of Sir Joseph Bazalgette – the engineer who had been responsible for the building of the London sewage and clean water systems (Halliday 1999) – the British press carried stories about the heroic efforts of Bazalgette and his importance in saving many lives. Both Koch's microbiology and Bazalgette's engineering had profound effects on late Victorian thinking. The threat of cholera was real and it had been a constant and recurring problem which had made a deep impression on the public (Hamlin 2009). The discovery that there was apparently an identifiable cause of pathology (in the form of the bacteria) and that there were the means of protection (clean water and sewage disposal) were unsurprisingly very significant. (Carter 2003, Hamlin 2009, McMichael 1999). Seductively appealing as the simple ‘cause – effect – protect’ argument is (Hanlon *et al*. 2012), there are several issues which are important to clarify.

First, the aetiology and action of bacterium**,** including cholera, is not as simple as ‘exposure equates to disease’ (Hamlin 2009). Aetiological pathways are very complex and are mediated by all sorts of factors from the epigenetics of the bacteria to the biological and social inheritance of the host. The understanding of pathogenesis and microbiology were slow and highly contested and, by the end of the nineteenth century, still very elementary (if compared to contemporary standards in, say, molecular biology); and yet they reinforced simple cause and effect ideas (see Carter 2003). It is also worth noting that the targeted diseases were not well understood scientifically either (Hamlin 2009; Holloway 1964). Even ideas which we now recognize as aligning with contemporary medical thinking were neither well‐articulated nor readily accepted. So, for instance, when John Snow proposed measures against the cholera epidemic in 1854 London, he did not know what the biological cause of cholera was (Paneth 2004), and neither did the reformers who championed the British and European sanitary movements (Mackenbach 2009a, 2009b). In 1847, Sommelweis had suggested disinfecting hands to stop puerperal fever in Vienna hospitals, but the causes were only hypothesised, and largely incompatible with the then contemporary medical thinking (Gillies 2005b, Russo and Williamson 2007, 2011). And the public were not particularly receptive either. William Tenant Gairdner, the first Professor of Public Health at Glasgow University and the first Medical Officer of Health in that city, ruefully observed to his students that:

certain leading articles in several of the London papers – the *Times* and *Spectator* for instance – wonderfully clever and smart leading articles, shewing [sic] as beyond all doubt, that there was not and never had been, any such thing as contagion at all; that it was all a fiction of the medical mind, devised long ago to frighten people, and embarrass commerce (Gairdner, 1862: 208).

Contagion jostled with miasma theory – the theory that disease originated from bad air – and miasma was supported by luminaries like Bazalgette himself as well as Florence Nightingale and Edwin Chadwick. In mid‐nineteenth century no one knew in a ‘contemporary scientific sense’ what was right or wrong. The eventual acceptance of germ theory was a significant step socially as well as scientifically and in simplified form remains with us.

Second, the political controversies and outright opposition to the public works like sanitation and sewage disposal show that there was considerable resistance to what might now appear to be obvious. It was certainly not a quick fix. Detailed historical scholarship reveals that in the nineteenth century public health improvement was a long slow war of attrition against vested interests of all kinds (See e.g., Chave 1958, Halliday 1999, Hamlin 1998, Jackson 2014, Johnson 2008, Porter 1998, 2009, Venters 2015). Progress was slow and hard won. *The Economist* declared in May 1848 for example:

Suffering and evil are nature's admonitions; they cannot be got rid of; and the impatient attempts of benevolence to banish them from the world by legislation […] have always been more productive of evil than good. (Quoted in Halliday 1999: 61)

A few years later *The Times* carried a leading article stating:

The nation […] [is] little disposed to endure a medical tyrant […] we prefer to take our chance of cholera and the rest than be bullied into health […] (The Times, 1st August 1854: 8).

Winning over the hearts and minds of a sceptical press and public, cost conscious politicians and a conservative medical profession took decades.

Third, the historical record reveals that those interventions which are now recognised to have been effective, worked on complex multi‐levels with multiple mechanisms. They targeted what today could be referred to as the structural determinants and social practices related to health. The power of this broad social approach, as initially championed by William Duncan the first Medical Officer of Health in Liverpool (Frazer 1947) and John Snow in relation to cholera (Johnson 2006), both of whom had a clear sense of broad social causes, gradually lost its voice as the century proceeded and as germ theory gained ascendancy.

By the end of the nineteenth century, it had come to be realised that by protecting people from certain microorganisms and by providing clean water and removing sewage public health could achieve a lot. These public health interventions have saved many thousands of lives in the UK and elsewhere, and they improved the quality of life for countless other people. It is worth noting too that such basic interventions are effective still today, notably in places where social inequalities are still very high (as for instance in Brazil, see e.g. Barreto and Aquino 2009, Barreto *et al*. 2010). But it is a complex historical story not a simple narrative and one where the distinction between aetiology and prevention easily gets lost.

## Smoking and risk

So far we have tried to dismantle the simple causal narrative according to which, in order to reduce the burden of non‐communicable diseases we only need to reduce the exposure to known risks, namely change a whole range of unhealthy behaviours. This simple narrative is built by analogy to disease causation for infectious diseases. However, this simple narrative does not stand up to close examination as we have shown by appealing to an analysis of contemporary policy document and to a (cursory) reconstruction of the history of public health in England. Our point is that mechanisms of disease and mechanisms of prevention are often different, and to design appropriate preventive intervention one needs to properly understand the social dimension of health and disease. We now illustrate what this ‘social practice’ approach involves, focusing on two examples: smoking and alcohol.

The discovery of the relationship between cigarette smoking and lung cancer was scientifically and medically enormously important (Doll and Hill 1950, 1952). For our purposes, though, it is the unintended consequence of the discovery that is of particular interest. In short, the recognition, of the links between smoking and ill health, has saved very many lives. However, it has also helped crystallise ideas about risk which reinforce the simple narrative referred to above. When Doll and Hill (1950) suggested a connection between smoking and carcinoma of the lung, they set in train an epidemiological industry seeking to find risks associated with different types of human conduct (Armstrong 1995). What is important to note, is that smoking is a behaviour – a social practice. Actually, the realisation that the behaviour ‘smoking’ *de facto* contributed to risk shifted the scientific problem from the realms of the biological to the realms of the psychological and social. However, the paradigm of prevention didn't shift with it; rather, it was reinforced. It gave rise to a new and ultimately unhelpful way of thinking about behaviour and risk, which through the second half of the twentieth century became a major driver in public health policy in the UK and elsewhere. The preoccupation with multiple proximal risk factors has actually eclipsed the idea of social causation which public health had generated in the early nineteenth century mentioned above (Frazer 1947, Gairdner, 1862). With the rise of germ theory and then the development of modern epidemiology, the idea of determining which individuals are at risk rather than determining the distribution of disease within and between populations became *de rigueur* (McMichael 1999). The underlying assumption is that if we can identify the risk, we can control it, because we have found the cause; behaviour change became a significant part of the solution.

Hill's (1965) account of causal inference, as well as the decades of subsequent research exploring the bio‐pathogenesis of tobacco and human health, allows us to understand with a high degree of certainty that there is a dose response relationship between exposure to cigarette smoke and lung cancer, as well as heart disease, emphysema, hypertension and stroke; we can advise smokers that if they persist in their habit, they are undoubtedly putting themselves in harm's way. However, it shouldn't be forgotten that Doll and Hill's first paper appeared in 1950 and the smoking ban in public places was implemented in England only in 2007. Out of 28 EU countries, only 17 have, as of today, comprehensive legislation about smoke‐free environments.

The behaviour of smokers, of governments, of industry and of advertisers didn't follow the science, not immediately, and not for many years, and in some cases still doesn't. What has been achieved has taken a long time and against considerable resistance from the tobacco industry (Michaels 2008, Taylor 1984). One of their tactics has been to sow confusion about the science. The industry has gone to considerable lengths to muddy the water about risk (Taylor 1984). Michaels (2008) has documented the ways in which efforts to control known hazards like tobacco have been thwarted and hampered by the creation of apparent scientific uncertainty about the evidence. By calling into question the evidence demonstrating the hazards or by demanding standards of absolute proof of cause and effect, the illusion of uncertainty and doubt about risk is created (Michaels 2008). The same insidious process is at work, for example, in rebuttals of minimum unit pricing to protect the population from very cheap alcohol and the smoke screens thrown up by the food industry on trans‐fatty acids, salt and sugar.

In the nineteenth century, vested interests were transparent in their opposition to reform on the grounds that it was a disutility to them (Checkland and Lamb 1982). In the twentieth century vested interests have become much less nakedly obvious. This is an important part of the story as industrial vested interests have been very good at supporting the simple narrative and placing the responsibility on consumers to change their behaviour. The alcohol industry's support for sensible drinking (more of which below) or pro‐smoking groups’ emphases on freedom of choice being good examples. Just as with attempts at public health and social reform in the nineteenth century, vested interests of various kinds seek to exert political influence in order to prioritise commercial interests above those of the health of the public. The political promotion of commercial vested interests which supports the simple narrative is doubly insidious. It helps to create an environment which encourages people to aspire to change their individual behaviour but also creates an environment in which it is very difficult to do so. Information abounds, but it seems to say a whole lot of contradictory things. The idea that responsible consumers can then make responsible choices is risible in the midst of all the apparent contradictions. That commercial interests are part of the causal pathway to disease and stand in the way of mechanisms of prevention is a political and commercial reality which sits comfortably with the simple narrative.

In sum, analysing smoking as behaviour, as a social practice, means not only to recognise the different contexts and reasons that encourage people to smoke or to quit, but also to recognise that people's smoking behaviour does not happen in a vacuum, but is instead part of a complex relation with commercials, industry, and governments, where vested interests are non‐negligible (Blue *et al*. 2016). Blue *et al*. (2016) demonstrate how, by using a social practice framework, it is possible to disentangle the many strands of the tobacco epidemic; how it was grown, manufactured, marketed, sold, taxed, consumed and the social meanings attributed to it and how they changed over time. The framework also helps to describe what the competencies were that social actors had to acquire in order to smoke and what the activity meant symbolically and culturally. Using an historical perspective, they show how these interrelated elements changed as they transited across time and how effective and successful tobacco control consisted of breaking the links between the elements of the practice of smoking.

## Preventing the misuse of alcohol

To further illustrate the importance of separating cause and prevention and of using a social practice approach to do better prevention, we consider alcohol‐related diseases and the public health efforts to prevent excessive alcohol consumption. The conventional approach of alcohol policy, at least in the UK, assumes that alcohol consumption is a general unitary behaviour which carries a risk; further, it is assumed that if we can change that general behaviour – and therefore predict future changes in conduct – we will reduce the risk by reducing exposure to the ethanol – the pathogen (McMichael: 1999). Policy documents also tend to assume that the alcohol industry is neutral in all this and doesn't really have a role in the increasing morbidity linked to increased sales of alcohol (HM Government 2012). The alcohol industry portrays itself as having drinkers’ best interests at heart as it wants them to drink responsibly (see http://www.portmangroup.org.uk/about).

It is assumed (and predicted) that if people can be persuaded to drink less, or to drink responsibly, the rates of a range of diseases of the liver, of certain cancers, of obesity, accidents, injury, and violence will decline. Generally speaking, the interventions that are traditionally deployed follow precisely the predictive approach based on the simple narrative. Something is done – for instance an education campaign, health warnings on bottles or messages on beer mats. This is done in the expectation that these interventions will reduce the individual behaviour of alcohol consumption, which in turn will have beneficial health and public order effects.

The problem with this approach is that it treats alcohol consumption, the causal pathways to disease(s), and the mechanism of preventing overconsumption as being the same. Following the simple narrative, one may expect that preventive strategies will work at some point in the future. Such expectation is based on the corresponding aetiological model where it ‘suffices’ to block or reduce the exposure to the pathogen.

But how can we predict behaviour on the basis of our knowledge of pathology in the liver, or of the psychoactive properties of alcohol? We cannot: these are not grounds for a prediction about behaviour or behaviour change at all. We are looking at, and trying to measure, the wrong thing (Pawson 2006). The focus should be, instead, on the social practices of alcohol consumption (Blue *et al*. 2016). Alcohol consumption is a practice which is clearly a part of the aetiology of alcohol‐related diseases. However, if we focus only on the biological pathogen (ethanol) and assume that the behaviour will take care of itself by persuasion to drink responsibly, we will never really understand how best to intervene to change it. We will miss a fundamentally important part of the socio‐economic‐behavioural mechanisms involved (Kelly *et al*. 2014).

Patterns of alcohol consumption among different groups, communities, age groups, occupations, even families and friendship circles are examples of highly variegated social practices, not the same general behaviour. The alcohol industry knows this, and segments the population for advertising and marketing purposes precisely in this way (Hastings 2009). Alcohol consumption and patterns of behaviour associated with its consumption vary greatly between different social groups (MacAndrew and Edgerton 1969; Mass Observation 2009). Drinking alcohol is *not* a single behaviour, but rather a whole range of quite distinct practices about which the human actors involved are deeply knowledgeable. What is drunk, how much is drunk, what goes on as the drinking takes place, the degree to which displays of intoxication are encouraged, tolerated, ignored or discouraged, are all highly nuanced features of the micro social environments where drinking occurs. There is also a complicating factor too in that some people are not able to adapt their alcohol consumption according to the different social spaces and habitually become very intoxicated. This behaviour tends to attract the label of problem drinking or alcoholic or some other usually negative and stigmatising definition. Of course, in certain ‘life worlds’ (Kelly *et al*. 2014) this type of behaviour will be shared and sanctioned by compatriots or peers, who also value frequent excessive intoxication over other social norms.

These different settings or contexts and what goes on in them are social structures which constrain interactive possibilities in delimited ways. The structures also enter human consciousness because the social actors involved have a degree of awareness of where they are, and of the social norms and expectations of the particular life world in which they find themselves. They attribute meaning to their own actions and the actions of others. They will have a sense of themselves existing in that particular life world, of having a sense of who and what they are, and what and why they are doing what they are doing (Giddens 1984). Instead of pursuing the explanation of alcohol consumption in terms of single cause‐effect relationship, where, most of the time, causes are inferred on the basis of bio‐chemical factors, we need to pose a different set of questions: about why the social actors involved in the interaction are there, what expectations they bring with them to the social setting and the skills and competencies they have to do the drinking successfully, what material infrastructures facilitate the practice and what meanings are generated about what is going on, etc. For each of the actors involved, or each of the groups, there will be an idiosyncratic configuration of reasons as to why they do the drinking behaviour that they do in the way that they do, which reflect their social competence, the available materials and places, what it means to them, the practices they habitually use and their connectedness with other social worlds and social norms.

Patterning is important because the total levels of alcohol consumption of both individuals and populations are products of the patterning. So trying to predict levels of consumption and the possibilities of change on the basis of single variables like psychological state, or socioeconomic background or even price, is not on its own going to do the explanatory heavy lifting; still less will knowledge of the aetiology of liver disease do so. A more systemic understanding of the social practices constituting alcohol consumption is what is needed. How is the practice sustained? What are the technologies and materials that facilitate it and how are these controlled and by whom? What are the competencies required of the social actors to engage effectively in the different types of social action involved and what are the meanings which the actors ascribe to what they are involved in (Shove *et al*. 2012)?

There is another very important dimension to consider. Meaning and interpretation are important, but so too are the reactive and responsive nature of human interaction, that is, what psychologists call the ‘automatic system’ (Marteau *et al*. 2011, Strack and Deutsch 2004). The automatic system refers to the fact that although humans are thoughtful reasoners some of the time, humans also do things in direct response to the immediate social and physical world around them as well. They take cues from their environment and respond accordingly. They take short cuts, their thinking is lazy, and they use habitual, stereotypical and prejudicial forms of reasoning that require little or no reflective thought. So even though they have the capacity to be reasoning and thoughtful, much of the time spent in human interaction is simply responding to immediate cues and signals and to the physical and social world as it appears to be in the here and now, what Giddens (1984: 6‐7, 90) called ‘practical consciousness’. Uncontrolled drinking is a case in point where the drinker is responding to certain cues including the subjective physical and psychological sensations associated with intoxication, rather than reflecting on and evaluating the social context in which they find themselves or the long term harm that might ensue.

Unravelling the social actions of specific types of drinkers in particular social settings (in a way analogous to what the industry selling alcohol does with market segmentation, Hastings 2009) allows us to get much closer to being able to see what might need to be changed in environments to affect behaviour in a way that knowing about the dose response relationship between litres of ethanol consumed and liver and other organ pathology does not.

Public health efforts to reduce or limit alcohol consumption focus though not only on the consumption of individual drinkers but on the population levels of consumption. In this regard we have witnessed a natural experiment since the late 1990s in the UK in which alcohol price relative to average wages has got cheaper and it has become much more widely available. Unsurprisingly, alcohol consumption has risen (See Booth *et al*. 2008 for a review of the relationship between price and consumption, and Marteau *et al*. 2015). This has led to recommendations for the introduction of minimum unit pricing (NICE 2010) a suggestion rejected by the Coalition Government. The economic arguments for minimum unit pricing are sound for those parts of the population that migrate to the cheapest forms of alcohol – the young and the very heaviest consumers (Meier *et al*. 2009). However, for the many other populations of drinkers, other tactics will be required if overall consumption is to be reduced and the understanding of the social practices of the many settings and contexts in which alcohol is consumed still require detailed sociological scrutiny. The scientific evidence here is as yet relatively sparse (though not we suspect the market research evidence!). A recent large scale scoping review found little evidence relating to the contexts of alcohol related behaviour (Hollands *et al*. 2013) and the research that has been done has focused on young people and/or binge drinking in public venues (Hughes *et al*. 2011, Maclean 2016).

In sum, in this case too, even a cursory analysis of the social practice of alcohol consumption shows how much the simple narrative is clearly inadequate to tackle public health problems effectively.

## Conclusion

In this paper, we have investigated the question of whether mechanisms of aetiology of disease are the same as the mechanisms to prevent disease. This is a timely question, in the light of the statistics about the increasing prevalence of some non‐communicable diseases.

We advance the view that mechanisms of aetiology and of prevention of NCDs are indeed very different. We build upon previous work (Kelly *et al*. 2014), in which it was suggested that mechanisms of health and disease are mixed, namely they involve biological as we well as socio‐eco‐psychological ones. Here, the argument goes one step further in that mechanisms of prevention should take this second category of factors more explicitly into account. We argue against the simple causal narrative by analysing the principles behind influential policy documents and by drawing attention to selected salient aspects of the history of public health in England. Further, we exemplify our claims using the cases of obesity, of smoking, and of alcohol consumption.

None of our arguments are to say that attempting to bring about public health improvement through interventions of various kinds such as information and education is a complete waste of time. Far from it. There is an evidence base that describes the cost effectiveness of some of these things (Owen *et al*. 2012); however, cost effective or not, their reach has remained limited and the inroads into the epidemics of NCDs has been less effective than it might have been if a broader approach, such as we have illustrated here with reference to social practices and alcohol consumption, had been applied.

We make the case for conceptualising the consumption of alcohol like this. But food consumption and taking exercise can also be conceptualised as social practices. We argue that by doing this it is possible to disassemble elements of the practices in ways that permit different solutions to preventing the epidemics. Central in this is the role of the industries which supply, advertise and manufacture the products. Also crucial to the new prevention will be a recognition that to be successful, prevention has to operate at multiple levels across time and space, that the complexity involved must be embraced in order to break the links between elements of the practices. Above all simple models of cause and effect drawn from a misunderstanding of the causes of infection and a poorly constructed account of nineteenth century public health history, must be dropped from policy.
